# Mapping Global Research on Microplastics in Dental Science: Scientometric Trends, Collaborations, and Future Directions

**DOI:** 10.1155/ijod/6506257

**Published:** 2026-10-05

**Authors:** Fran Espinoza-Carhuancho, Ivan Calderon-Cortez, Mabel Huaman-De la Cruz, Felipe Lozano-Castro, Justiniano Sotomayor Camayo, Victor Velasquez-Rimachi, Frank Mayta-Tovalino

**Affiliations:** ^1^ Neuroscience Metabolism Clinical and Health Efficacy Research Group (NEMECS) Universidad Científica del Sur Lima Peru cientifica.edu.pe; ^2^ EVIDENTIA Research Group Universidad Nacional Mayor de San Marcos Lima Peru unmsm.edu.pe; ^3^ Academic Department Faculty of Dentistry Universidad Nacional Federico Villarreal Lima Peru unfv.edu.pe; ^4^ Academic Department Faculty of Dentistry Universidad Nacional Mayor de San Marcos Lima Peru unmsm.edu.pe; ^5^ Academic Department Vicerrectorado de Investigacion Universidad San Ignacio de Loyola Lima Peru usil.edu.pe

**Keywords:** bibliometrics, dentistry, microplastic, nanoplastics

## Abstract

**Aim:**

To analyze the global scientific production on microplastics in dentistry by evaluating publication performance, impact indicators, collaboration networks, thematic evolution, and core publication sources.

**Materials and Methods:**

A scientometric study was conducted based on publications indexed in the Scopus database. Publications indexed between January 1, 2020 and January 17, 2026, were retrieved in accordance with the Reporting and Measurement Items for Bibliometric or Scientometric Studies in Health Sciences (RAMIBS) guideline. We examined various document types written in all languages; records were exported from Scopus in CSV format and analyzed using SciVal for bibliometric indicators. We also analyzed the records that were exported from Scopus in CSV format and analyzed using SciVal (Elsevier) and R version 4.5.2 in RStudio with the bibliometrix package for data cleaning, normalization, and bibliometric analyses. Variables examined included author, affiliation, country, scholarly output, citations, views, journal quartiles, distribution of themes according to Bradford’s law, collaborative networks, thematic factor analysis, and the temporal evolution of the themes studied.

**Results:**

The analysis identified 75 publications from 45 sources with an average citation per article of 10.7 citations per article. These publications had an average age of 2.6 years, were written by 317 authors, and demonstrated a total rate of 21.3% in terms of the number of international collaborations. There was a predominance of original articles (42) and review articles (28) among these publications, which were published in core journals following the distribution of publications according to the Bradford law of distribution. The authors operated in relatively sparse collaboration networks with little local collaboration with other authors, while at the same time international collaboration showed a relatively dispersed network, with India occupying a prominent position based on country‐level collaboration indicators. Thematic analysis identified co‐occurring themes linking microplastics, dentistry, environmental pollution, and carbon footprint.

**Conclusion:**

Research on micro‐ and nanoplastics (MNPs) in dentistry is an emerging and interdisciplinary field with relatively limited international collaboration.

## 1. Introduction

Today, plastics are deeply integrated into modern life, offering significant benefits in various fields [1]. However, their indiscriminate production and disposal have caused serious environmental impacts, making them a major global problem [2]. Plastics, especially microplastics, are present in virtually all terrestrial and marine environments, posing a growing risk to wildlife, the balance of ecosystems, and possibly human health [3, 4].

In recent years, micro‐ and nanoplastics (MNPs) have raised growing concern due to their effects on the environment and health. Dentistry is no exception to this situation, as it uses polymeric materials and oral hygiene products that may contribute to this exposure [5]. Furthermore, the presence of microplastics has been linked to various potential risks to the human body [6]. Recent research indicates that these particles can be ingested or incorporated into oral tissues, promoting the onset of inflammatory responses, oxidative stress, and possible genotoxic effects [7].

Plastics are widely used in modern dentistry, both in single‐use clinical supplies and long‐lasting restorative materials. Given the global increase in plastic waste, there is growing concern about dentistry’s potential contribution to pollution from microplastics and nanoplastics, which could pose a risk to patients and the environment [8, 9]. Dental practice generates plastic waste through the extensive use of single‐use materials and polymer‐based products [8]. Plastic particles generated by dental appliances include those produced by mechanical wear, changes in the pH and temperature of the mouth, and the dissolution of acrylic and other dental materials. Therefore, dental materials erode over time, losing their structural integrity and eventually releasing additional monomer(s) or other harmful substances that may adversely impact human health or the environment [9].

Microplastic exposure may also occur through inhalation. Dental materials and devices may release particulate matter through mechanical wear and degradation during clinical use. Microplastic exposure may also occur through inhalation. Dental materials and devices may release particulate matter through mechanical wear and degradation during clinical use. Clear aligners, dentures, resin‐based restorations, and other polymer‐based devices may therefore represent potential sources of microplastic exposure in the oral environment [10].

Bibliometrics is based on the statistical analysis of the published scientific literature on a specific topic, allowing for the identification of the countries, institutions, authors, and journals that lead academic output in each field. This tool is key to analyzing the development and dynamics of a line of research and identifying patterns, emerging trends, and collaborative networks [11, 12]. It also helps guide future research, strengthen ties among researchers, and evaluate the impact and scientific reach of publications [13].

No previous scientometric studies have evaluated microplastics in dentistry. Given the emerging nature of this field, heterogeneous publication patterns and limited international collaboration were expected. Thus, the objective of this study was to characterize global scientific production on MNPs in dentistry by examining publication trends, research impact, collaboration networks, thematic patterns, and core publication sources.

## 2. Materials and Methods

A scientometric study was conducted using bibliographic records indexed in Scopus. The search was performed on January 17, 2026, covering publications from January 1, 2020 to January 17, 2026. Reporting followed the RAMIBS guideline [14].

### 2.1. Search Strategy

The literature search was conducted in the Scopus database on January 17, 2026. Records published between January 1, 2020 and January 17, 2026, were considered eligible for inclusion. The following search query was used, employing Boolean operators and truncated terms: TITLE‐ABS (“microplastic^∗^” OR “micro‐plastic ^∗^” OR “plastic debris” OR “plastic particle ^∗^” OR “plastic waste” OR “plastic pollution” OR “nanoplastic ^∗^” OR “synthetic polymer ^∗^” OR “environmental plastic ^∗^” OR “plastic contaminant ^∗^” OR “plastic fragment ^∗^”) AND SUBJAREA (DENT) AND PUBYEAR > 2019 AND PUBYEAR < 2027. Since researchers studying microplastics in dentistry often do not use standardized terminology for these materials, we deliberately allowed for wider search terms to retrieve additional relevant publications. Retrieved records were manually screened to confirm their relevance to microplastics or nanoplastics in dentistry. The search strategy was restricted to title and abstract fields, while author keywords were subsequently used for bibliometric and thematic analyses.

The reliability of the search methodology and the focus on both specificity and sensitivity was strengthened through a second opinion on the searches conducted. Both of these were performed by independent reviewers with extensive experience in bibliometrics; the results of these reviews were then evaluated against the pre‐existing criteria established prior to the screening.

### 2.2. Selection Criteria

Eligible records included all publications indexed in Scopus with a classification under the dentistry subject area and published from January 2020 through January 17, 2026. No restrictions were imposed on publication language or document type, provided that the records met the predefined eligibility criteria. Publications with incomplete citation information, duplicate records, or not relevant to dental practice were removed. Scientometric studies do not limit the type of study design; therefore, no limitations were placed upon this population of articles. There were also no restrictions based upon language. No formal translation of any publications was conducted; however, the scientometric analysis was primarily based on bibliographic citation information obtained from Scopus, including titles, abstracts, keywords, authorship, institution of author, and citation history. Language‐specific differences were therefore not translated or interpreted at the full‐text level (Figure 1).

**Figure 1:**
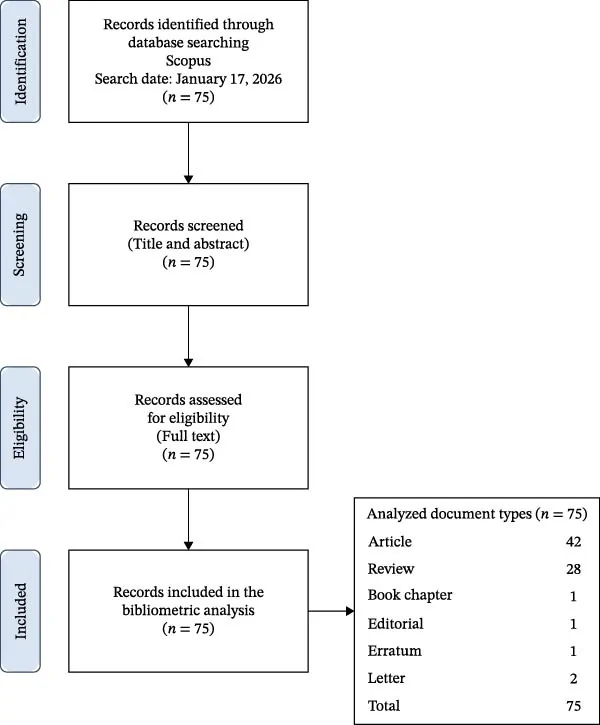
Flowchart.

### 2.3. Database Selection

Two reviewers independently screened all records retrieved from Scopus to determine their eligibility for inclusion. Disagreements between reviewers were resolved through discussion and consensus; when consensus could not be reached, a third reviewer was consulted to make the final decision. Scopus was selected as the sole bibliographic database because the study incorporated SciVal as the principal platform for the assessment of scientific output, citation impact, international collaboration, and research performance. SciVal is a research analytics tool provided by Elsevier that uses bibliographic data obtained from Scopus. Using Scopus as its primary database has provided consistency between the two ways of measuring bibliometric performance and therefore the analytical results obtained through SciVal. The intention of this project was to use as many common sources as possible to obtain a level of methodical consistency to allow for the most accurate comparison of the results obtained. However, one limitation of this study was the potential for the use of a single database to create coverage/bias on specific areas related to your research area in the database.

### 2.4. Data Processing and Bibliometric Workflow

The bibliographic records retrieved from Scopus were exported and processed for bibliometric analysis. SciVal was used to assess scientific output, citation performance, international collaboration, and research impact, whereas R (version 4.5.2 in RStudio) was used for data cleaning, data organization, normalization, and supplementary bibliometric analyses.

The data‐processing workflow consisted of sequential steps, including data import, verification of bibliographic information, identification and removal of duplicate records, standardization of author and institutional information, and normalization of keywords. Author names and institutional affiliations were reviewed and standardized to minimize inconsistencies resulting from differences in spelling, abbreviations, and formatting. Keywords were manually reviewed and normalized to consolidate equivalent terms, spelling variants, singular and plural forms, and differences in hyphenation. Terms referring to the same concept were grouped under a standardized keyword to minimize artificial fragmentation of the thematic structure. The keyword normalization procedure was documented in a thesaurus file and is provided as Supporting Information.

The bibliometric analysis included annual scientific production, citation performance, author productivity, journal productivity, country contributions, institutional contributions, international collaboration, Bradford’s law analysis, and keyword‐based thematic analysis. SciVal indicators were derived directly from the Scopus‐indexed dataset to maintain consistency between the bibliographic records and the corresponding bibliometric metrics.

For the bibliometric analyses performed in RStudio, the cleaned and normalized dataset was used as the analytical corpus. Author, institutional, country, and keyword information was analyzed according to the bibliographic relationships identified within the dataset. Network and keyword analyses were interpreted as descriptive representations of co‐authorship and keyword co‐occurrence patterns rather than as evidence of causal or conceptual relationships.

All data processing and analytical procedures were documented to improve methodological transparency and reproducibility.

### 2.5. Data Analysis

Bibliometric data were analyzed using R version 4.5.2 in RStudio with the bibliometrix package and SciVal (Elsevier). Scientific production and impact were assessed using publication counts, citations, citations per publication, total views, and views per publication. Author, institutional, country, and journal productivity were also evaluated. Journal concentration was assessed using Bradford’s law. International collaboration was analyzed through country‐level co‐authorship networks. Temporal trends were evaluated using annual scientific production, cumulative growth, and research life‐cycle analyses. Thematic structure was explored using keyword‐based conceptual analysis in bibliometrix. Author keywords were normalized before analysis, and the conceptual structure was visualized according to the resulting thematic dimensions. All analyses were descriptive, and no predictions of future publication growth or saturation were made.

## 3. Results

A total of 75 documents published between January 1, 2020 and January 17, 2026, were identified. Scientific production peaked in 2024, followed by lower output in subsequent years; however, the 2026 count should be interpreted cautiously because the search covered only part of the year. Despite this decline, the retrieved publications accumulated a mean of 10.7 citations per document, indicating scientific visibility within this emerging research field. The average document age was 2.6 years, confirming that research on microplastics in dentistry is a recently emerging field. There were also large numbers of authors who participated in producing these articles; the 75 publications involved 317 authors, with an average of 4.3 co‐authors per publication. International co‐authorship was present in 21.3% of publications, while 9 publications were single‐authored. The predominant types of articles published were original research articles (42) and review articles (28). Original research articles predominated, followed by review articles (Table 1).

**Table 1:** Main information.

Description	Results
Timespan	2020:2026
Sources	45
Documents	75
Annual growth rate %	−12.6
Document average age	2.6
Average citations per doc	10.7
References	673
Author’s keywords	267
Authors	317
Authors of single‐authored docs	9
Single‐authored docs	9
Co‐authors per doc	4.3
International co‐authorships %	21.3
Article	42
Book chapter	1
Editorial	1
Erratum	1
Letter	2
Review	28

The most productive authors showed heterogeneous levels of citation impact and publication visibility. Lobat Tayebi had the highest average views per publication (58.5) and citations per publication (63) among the leading authors. Nicolas Martin and Steven Mulligan each had 36 views per publication and 19.5 citations per publication. Theodore Eliades and George Eliades each had 16.5 views per publication and 14 citations per publication. Among authors with a single publication, Zsuzsanna Czégény had the highest views per publication (88), whereas Farahnaz Nejatidanesh had the highest citations per publication (25) (Table 2).

**Table 2:** Top 10 most productive authors.

Author	Affiliation	Country	Scholarly output	Views count	Views per publication	Citations per publication
Eliades, Theodore	University of Zurich	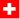 Switzerland	2	33	16.5	14
Eliades, George	National and Kapodistrian University of Athens	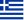 Greece	2	33	16.5	14
Tayebi, Lobat	Old Dominion University	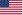 United States	2	117	58.5	63
Martin, Nicolas	University of Sheffield	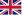 United Kingdom	2	72	36	19.5
Mulligan, Steven	University of Sheffield	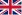 United Kingdom	2	72	36	19.5
Siswomihardjo, Widowati	Gadjah Mada University	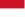 Indonesia	1	16	16	0
Zlámalová‐Gargošová, Helena	Brno University of Technology	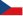 Czech Republic	1	7	7	13
Czégény, Zsuzsanna	Research Centre for Natural Sciences	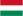 Hungary	1	88	88	19
Nejatidanesh, Farahnaz	Isfahan University of Medical Sciences	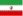 Iran	1	44	44	25
Sonnesen, Liselotte	University of Copenhagen	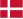 Denmark	1	15	15	5

The distribution of publications across journal quartiles varied over the study period. Q3 showed the largest increase in publication output in 2024, whereas Q2 reached its highest output in 2025. Publication counts were lower in 2026, which should be interpreted cautiously because the year was incomplete. Q1 journals showed a relatively stable contribution throughout the study period (Figure 2).

**Figure 2:**
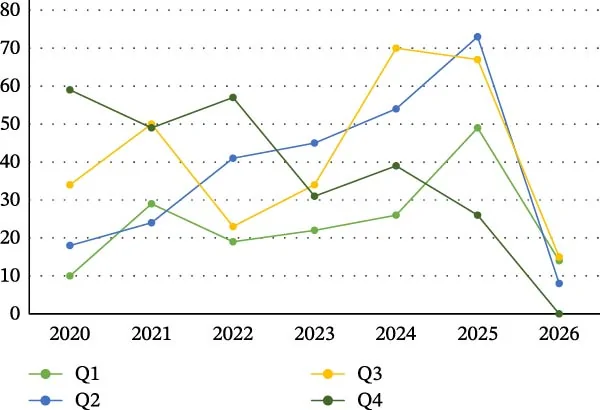
Impact by quartile of scientific publications.

Bradford’s law indicates that there is a strong clustering of scientific output within a limited number of journals, specifically the Journal of Thermal Analysis, British Ceramic Journal, Silicaty Journal, International Journal of Thermophysics, Journal of Materials Science, and Sri Lankan Journal. Bradford’s law identified that most publication sources within the analyzed corpus represent the first Bradford zone. Core status was determined by publication concentration and does not imply dentistry‐specific scope (Figure 3).

**Figure 3:**
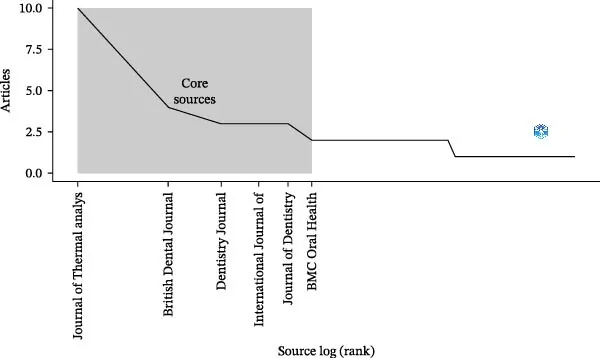
Core sources by Bradford´s law.

International collaboration was assessed through country‐level co‐authorship networks. Countries were considered connected when authors affiliated with the respective countries co‐authored at least one publication within the analyzed corpus. Using statistical network attributes—the total number of publications, total number of collaboration relationships, total link strength of all collaborators, degree centrality, and betweenness centrality—the collaborative network was characterized. Degree centrality defined how many direct relationships were established by each country in the collaborative network, and betweenness centrality identified countries in potential bridging positions between less connected countries/groups. Total link strength was used as an indicator of the overall strength of a country’s collaborative relationships. The network structure was visualized in Figure 3, while quantitative network metrics were used to support the interpretation of the most prominent countries within the international collaboration network. These measures were interpreted descriptively and were not considered evidence of causal influence or scientific leadership. Line width represents the strength of the collaborative relationship (Figure 4).

**Figure 4:**
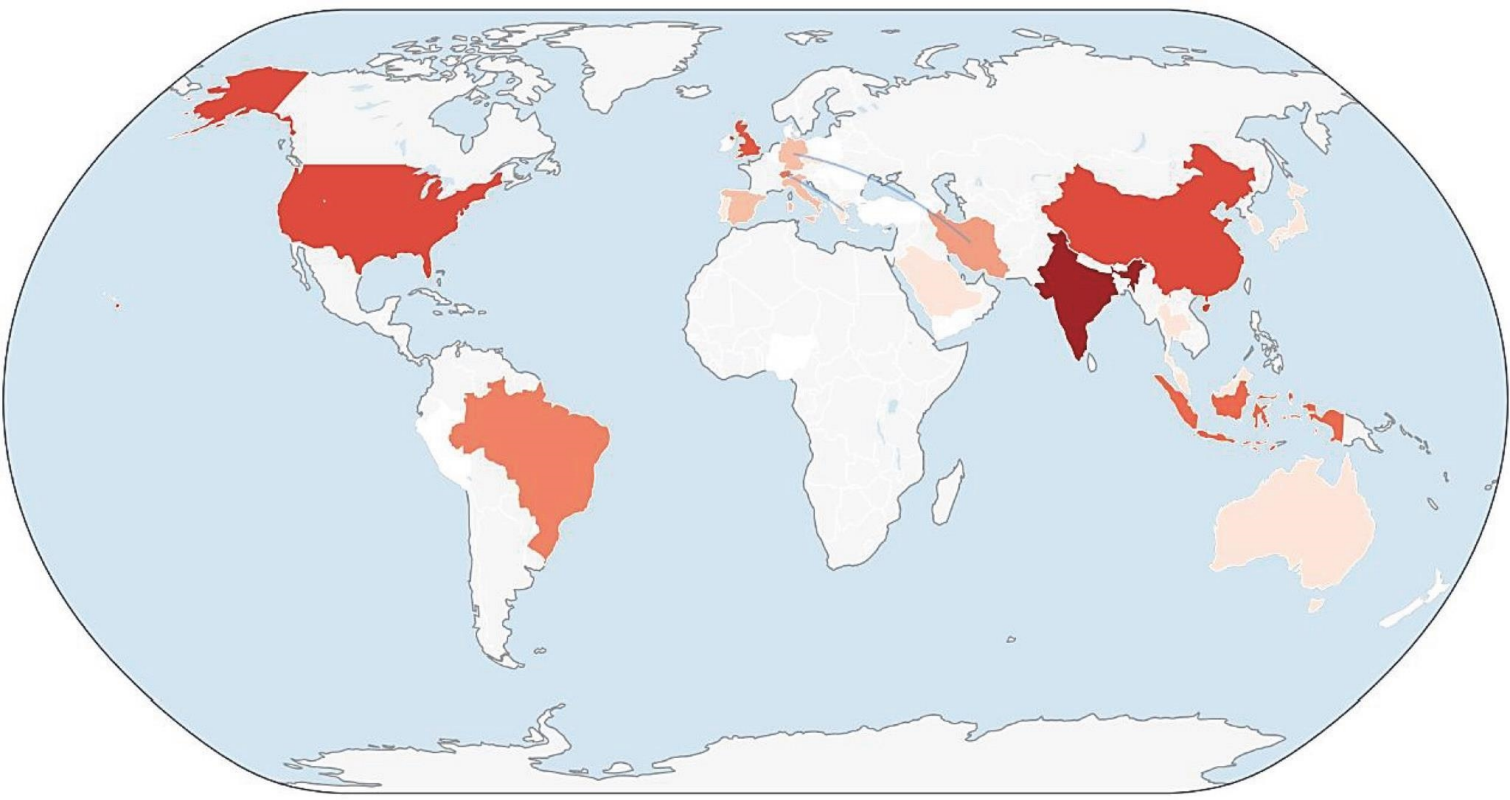
Country collaboration map. Color intensity represents country‐level publication output, with darker shades indicating a higher number of publications. Lines represent international co‐authorship links, and line width represents the strength of collaborative relationships.

The conceptual map showed the co‐occurrence of the terms “carbon footprint,” “dentistry,” “microplastics,” and “environmental pollution” within the same thematic cluster. Their proximity in the factorial space indicates shared co‐occurrence patterns within the analyzed corpus. These findings describe the thematic structure of the literature and should not be interpreted as evidence of causal or conceptual relationships between the identified terms (Figure 5).

**Figure 5:**
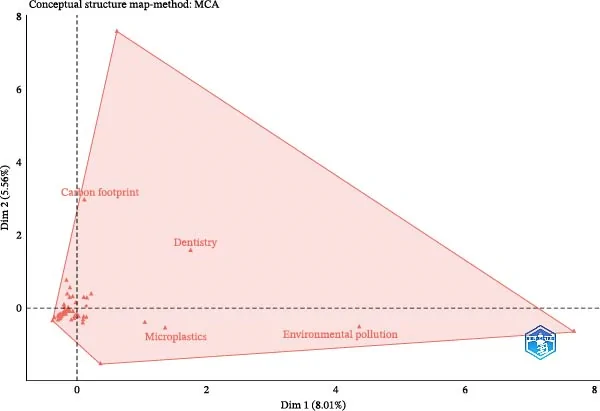
Factorial analyses.

The cumulative growth curve showed an overall increase in scientific production during the study period, indicating sustained growth in the number of publications on MNPs in dentistry. However, the available bibliometric data do not support extrapolation of the publication trajectory to future years or the prediction of a specific saturation point. The research life‐cycle analysis indicated that the highest level of author productivity was observed in 2024, followed by lower observed output in subsequent years; however, the 2026 estimate should be interpreted cautiously because the search covered only part of the year. Therefore, the observed trends should be interpreted as descriptive patterns within the analyzed period rather than as predictions of future publication volume or research saturation (Figure 6).

**Figure 6:**
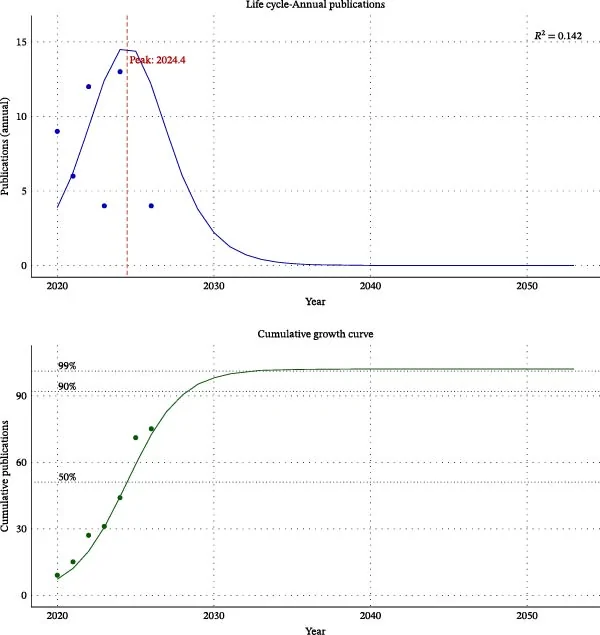
Life cycle and cumulative growth curve.

## 4. Discussion

Microplastics have recently become a serious environmental pollutant, and dental practices create microscopic particles/nanoplastic particles as a result of handling and/or wearing of these products; therefore, these particles could then be potentially released into the environment [15]. Research on the environmental and health implications of MNPs has primarily developed within environmental, materials, and toxicological sciences, while its application to dentistry has emerged more recently. There is a significant number of dental materials that contain polymers in their structure and use, so there are many ways for the use of these materials in clinical practice to result in exposure for dentists and patients. This leads to increased concern over the potential for MNP to have negative effects on human health [16].

In our study, Bradford’s law was applied to evaluate the distribution and concentration of publications across journals within the retrieved Scopus‐indexed corpus. Journals were ranked according to the number of publications and classified into Bradford zones according to their relative contribution to the total scientific output. The journals identified within the first Bradford zone were considered core publication sources of the analyzed literature. Importantly, the identification of a journal as a Bradford core source was based exclusively on its publication frequency within the study corpus and did not imply that the journal was dentistry‐specific. Given the interdisciplinary nature of MNPs research, journals from dental, biomedical, materials science, environmental science, and related disciplines were considered eligible for the analysis.

Through our review of the literature on microplastics in dentistry, we found that between 2020 and 2026, there were 75 published documents authored by 317 researchers who published in 45 journals about microplastics in dentistry. These researchers had an average of more than four authors on each of their documents, the documents included an average of 4.3 co‐authors per publication, reflecting a predominantly collaborative authorship structure; however, their work has very limited international reach outside the region of the authorship. Although the average number of citations (10.7) per document is high, the mean citation count was 10.7 citations per document, indicating measurable citation visibility within the analyzed corpus. Most of the publications were in the form of original research articles and reviews, which demonstrated a balanced effort between generating evidence and conducting critical reviews of evidence. By comparison, Viru‐Vasquez et al. reported an extremely large body of published works (239 total publications) during the last decade—an increase of 54.4% each year—whereas their work involved the collaboration of over 1000 authors from many different countries outside of Venezuela. Taken together, these data suggest that environmental research on microplastics is more established and expanding, whereas in the field of dentistry, it represents a more recent and specialized area that is still in the process of gaining strength [17].

Lobat Tayebi showed the highest views per publication and citations per publication among the leading authors in the analyzed corpus. In Europe, researchers such as N. Martin and S. Mulligan (University of Sheffield, UK), T. Eliades (University of Zurich, Switzerland), and G. Eliades (National and Kapodistrian University of Athens, Greece) displayed very good and balanced levels of citations and productivity. Farahnaz Nejatidanesh had the highest citations per publication among the single‐publication authors listed in Table 2. The results of this research indicated a wide geographical distribution of different studies, although the studies were narrower in scope than previous research. Conversely, R.C. Thompson (UK), who was identified as the most influential author by Qin et al. in their review of global research on microplastics, has produced a large number of publications and has amassed many thousands of cumulative citations in addition to being one of a number of other European and Asian researchers in the field of ecology and environment with a high citation record. In this regard, both studies demonstrated that research on microplastics is collaborative and transnational; however, while leadership in the environmental field is characterized by massive and well‐established global influence, in dentistry, the impact is developing within a more specific field that is undergoing scientific expansion [18].

Lou et al. depicted a very strong network of global partners where several countries worked together and remained in close and frequent communication with each other; the ties between these countries were also very intense and multiple (especially with regard to countries like China–United States, Australia–United Kingdom, and the United States–Canada). Therefore, it is reasonable to conclude from this description that the overall pattern of global cooperation was a single, unified, and continuous structure. On the other hand, our study revealed that the level of vibrancy of the network we analyzed is considerably lower than that of Lou’s Network, with a substantial proportion of the contingent partners that were the subject of this study not having connections to other countries or only having direct relationships via isolated bilateral partnerships. In the collaboration network across countries, India demonstrated the greatest degree centrality (as well as the greatest total link strength), signifying that there were numerous connections that interconnected India and all the regions within the observed collaboration network. While the collaborations exhibited a very high level of collaborative strength and proximity of connectivity to the rest of the regions, the overall density and structural integration of these connections are considerably less than those documented by Lou et al., demonstrating a lower level of collaboration and integration of the international community relative to Lou et al. [19].

The study of the sources using Bradford’s law illustrated the concentration of scientific production in a limited number of journals. For example, Bradford’s law identified six core publication sources within the first Bradford zone, reflecting concentration of publication output within a limited number of journals: Journal of Thermal Analysis, British Ceramic Journal, Silicaty Journal, International Journal of Thermophysics, Journal of Materials Science, and Sri Lanka Journal. All other publications were produced across many other journals with low frequency. This finding is consistent with the characteristics of the model of core‐periphery. In contrast, Yildirim et al. found that the concentration of publications in high‐impact environmental journals was greater. The three most productive high‐impact journals were Science of the Total Environment (52 publications), Environmental Pollution (47 publications), and Journal of Hazardous Materials (45). There were also two other high‐impact journals with a significant number of publications: Water Research and Chemosphere, which had 30 and 29 publications, respectively. These journals not only accounted for a higher volume of articles but also achieved citation averages exceeding 20 per paper, reflecting a higher level of impact and international recognition compared to the more specialized scope of our study [20, 21].

This study assessed recent studies on microplastics in dentistry to provide an updated summary of how microplastic research in dentistry has progressed. The type of evaluation completed in this assessment was an evaluation based on several metrics, which include publication count, the progression of publication activity throughout time, citation counts or citation impact measures for the published materials, as well as collaborative patterns of authors/institutions. First, the exclusive use of Scopus may have resulted in the omission of publications indexed exclusively in other databases. Second, citation‐ and view‐based indicators may be influenced by publication age, database coverage, and disciplinary differences and should not be interpreted as direct measures of research quality. Third, the analysis was limited to publications indexed up to January 17, 2026, and the 2026 publication count was therefore incomplete. Finally, keyword normalization and thematic interpretation may be influenced by indexing practices and author terminology.

## 5. Conclusion

Research on MNPs in dentistry remains an emerging and interdisciplinary field, with 75 Scopus‐indexed publications produced between 2020 and January 17, 2026. Original articles and reviews were the dominant forms of scientific output, while a few core sources of publication were identified through Bradford’s law analysis. Only 21.3% of publications had international co‐authorship, indicating that scientific research is not yet fully integrated worldwide. Analysis of the themes demonstrated that the interdisciplinary nature of this area includes dentistry, microplastics, environmental pollution, and carbon footprint. Further empirical research and increased international collaboration can support the continued growth of this field.

## Author Contributions

**Justiniano Sotomayor Camayo**, **Fran Espinoza-Carhuancho**, **Felipe Lozano-Castro**, **Ivan Calderon-Cortez**: conceptualization, data collection – history, search strategy, and selection. **Victor Velasquez-Rimachi**: methodology, project administration – methods, project administration, and administrative processes. **Justiniano Sotomayor Camayo**: writing – original draft – introduction. **Fran Espinoza-Carhuancho**, **Mabel Huaman-De la Cruz**, **Frank Mayta-Tovalino**, **Victor Velasquez-Rimachi**: formal analysis, visualization, writing – review and editing – results, tables and figures, conclusions, and critical review of the manuscript.

## Funding

No funding was received for this manuscript.

## Ethics Statement

Not applicable, as this scientometric study used publicly available data from open‐access sources.

## Consent

The authors have nothing to report.

## Conflicts of Interest

The authors declare no conflicts of interest.

## Data Availability

The data resulting from the literature search can be made available upon a suitable request directed at the corresponding author. This ensures that the research process remains transparent and accessible for further study or verification.
